# Functional characterization of goose IRF1 in IFN induction and anti-NDV infection

**DOI:** 10.1186/s13567-022-01046-9

**Published:** 2022-04-04

**Authors:** Zhenyu Lin, Jie Wang, Nian Zhang, Jianshu Yi, Zhaofei Wang, Jingjiao Ma, Hengan Wang, Yaxian Yan, Kun Qian, Jianhe Sun, Yuqiang Cheng

**Affiliations:** 1 Shanghai Key Laboratory of Veterinary Biotechnology, Agriculture Ministry Key Laboratory of Urban Agriculture (South), School of Agriculture and Biology, Shanghai Jiao Tong University, Shanghai, 200240 China; 2 Jiangsu Key Laboratory of Zoonosis, Yangzhou University, No.48 East Wenhui Road, Yangzhou, 225009 Jiangsu China; 3 Ministry of Education Key Lab for Avian Preventive Medicine, Yangzhou University, No.48 East Wenhui Road, Yangzhou, 225009 Jiangsu China

**Keywords:** Goose, IRF1, IFN, innate immunity, NDV

## Abstract

Interferon regulatory factors (IRFs) play a key role in many aspects of immune response, and IRF1, IRF3, and IRF7 are positive regulators of IFN induction in mammals. However, IRF3, as the most critical regulatory factor in mammals, is naturally absent in birds, which attracts us to study the functions of other members of the avian IRF family. In the present study, we cloned goose IRF1 (GoIRF1) and conducted a series of bioinformatics analyses to compare the protein homology of GoIRF1 with that of IRF1 in other species. The overexpression of GoIRF1 in DF-1 cells induced the activation of IFN-β, and this activation is independent of the dosage of the transfected GoIRF1 plasmids. The overexpression of GoIRF1 in goose embryonic fibroblasts (GEFs) induced the expression of IFNs, proinflammatory cytokines, and IFN-stimulated genes (ISGs); it also inhibited the replication of green fluorescent protein (GFP)-tagged Newcastle disease virus (NDV) (NDV-GFP) and GFP-tagged vesicular stomatitis virus (VSV) (VSV-GFP). Our results suggest that GoIRF1 is an important regulator of IFNs, proinflammatory cytokines, and ISGs and plays a role in antiviral innate immunity in geese.

## Introduction

As a host’s first line of defense against exogenous insults, the innate immune system, which is broadly conserved in vertebrates, plays an important role in the host’s resistance to infections such as viruses, bacteria, and parasites. Pathogen-associated molecular patterns (PAMPs) are evolutionarily conserved molecular vital structures in pathogens, such as lipopolysaccharides, glycoproteins, proteoglycans, and nucleic acid motifs (RNA and DNA) [1]. Pattern recognition receptors (PRRs) located on the cell membrane or in the cell, which include nucleotide binding oligomerization domain (NOD)-like receptors (NLRs), toll-like receptors (TLRs), retinoic acid-inducible gene-I (RIG-I)-like helicases (RLHs), and several cytoplasmic DNA sensors, are proteins encoded by host germline genes that can recognize PAMPs [2–6]. The innate immune system is particularly critical for the host to detect invading pathogens and activate subsequent adaptive immunity [7]. The triggering of this system depends on the host’s PRRs to specifically recognize and bind to PAMPs and then activate related receptor-dependent signaling cascades to induce cytokine production [2]. Exogenous insults, such as bacteria, viruses, and parasites, are quickly recognized by the host's innate immune system, which then induces several immune mechanisms to resist invasion, including an interferon (IFN)-mediated antiviral response and an interleukin (IL)-mediated proinflammatory response [8, 9]. The type I IFN response is an effective defense of the host against viral infection [10].

Type I IFN is an important class of antiviral cytokines, including IFN-α, IFN-β, IFN-κ, and so on [11]. The induction of type I IFN is mainly controlled at the gene transcriptional level, and the interferon regulatory factors (IRFs) play central roles [12]. IRFs are transcription factors in IFN signaling pathways that play an important role in immune responses against bacterial or viral invasions [13]. So far, 11 members of the IRF family, IRF1 to IRF11, have been identified in vertebrates and 9 of them are expressed in mammalian cells (IRF1–IRF9) [14]. Compared with mammals, birds seem to naturally lack IRF3 and IRF9 [15], but the unique IRF10 can be found in them [16]. More importantly, it is widely accepted that a closely related IRF7, considered to be the major IRF in birds, complements the functions of IRF3, considered to be the critical IRF in mammals, and that other IRFs such as IRF1 may also compensate the functions of IRF3 to a certain extent [17–20]. In addition, both IRF10 and IRF11 have been identified in fishes [21]. The amino terminus of all IRFs contains a well-conserved DNA binding domain (DBD), which is characterized by five well-conserved tryptophan (Trp, W)-rich repeats, and the DBD contains a helix-turn-helix domain that recognizes specific DNA sequences [22]. The C-terminal amino acid of IRFs is less well conserved and contains an IRF-associated domain (IAD), which confers each IRF a unique function [23].

IRF1 is the first IRF family member found to be involved in the regulation of IFN [24]. Conservatively, the first 115 amino acids of the N-terminal of IRF1 contain a DBD with five highly conserved tryptophan-rich repeats that determines its transcriptional activity [25]. Mammalian IRF1 binds to the hexanucleotide unit in the positive regulatory domain 1 (PRD1) of the IFN-β promoter to regulate IFN expression [26]. Crystallographic studies further revealed that the 5′-GAAA-3′ sequence of PRD1 is the core sequence recognized by the DBD of IRF1 [27]. The conserved tryptophan cluster within the DBD was found to be critical in recognizing the core sequence. Three of the five tryptophan residues (W11, W38, and W58) fix the helix-turn-helix motif within the DBD to the major groove of the DNA, forming contact with the sugar-phosphate backbone and allowing the other four conserved amino acids of the DBD [Arginine (Arg, R) 82, Cysteine (Cys, C) 83, Asparagine (Asn, N) 86 and Serine (Ser, S) 87] direct contact to the core sequence [27]. Mammalian IRF1 plays an important regulatory role in antiviral innate immunity. After a viral infection, IRF1, which is significantly upregulated and expressed in cells, inhibits virus replication by regulating the production of IFN [28, 29]. IRF1 interacts with myeloid differentiation primary-response protein 88 (MyD88) to regulate the TLRs (TLR2, TLR3, TLR4, TLR7, TLR8)-dependent signal cascade and promote the production of IFN or IL to resist infection [30]. In addition, IRF1 has also been confirmed to be involved in the regulation of physiological or pathological processes, such as tumor immune surveillance, proinflammatory injury, immune system development, and autoimmune diseases [31].

The immune systems of birds and mammals are quite different, and birds lack several key immune genes [32]. IRF3, as one of the most critical IRFs in mammals, is naturally absent in poultry [33, 34]. Whether its absence leads to avian immune system deficiency remains to be studied. IRF1 plays a role in mammalian antiviral innate immunity, but its function in poultry needs to be more comprehensively studied. It is not clear whether avian IRF1 can compensate for the missing IRF3 function. Our previous study demonstrated that chicken IRF1 inhibits the replication of avian influenza virus (AIV) and Newcastle disease virus (NDV) by regulating the production of IFN-β and ISGs [35]. Duck IRF1 regulates the production of IFN-β by interacting with MyD88, effectively inhibiting virus replication [36]. However, the characteristics and functions of IRF1 in geese have not yet been elucidated. Chickens, ducks, and geese have different levels of resistance to AIV and NDV. As a key regulatory factor of the immune system, the functional difference of IRF1 may be the reason for the difference in antiviral levels of different birds. Therefore, it is necessary to conduct in-depth research on the function of GoIRF1 in antiviral innate immunity.

In the present study, GoIRF1 was identified and cloned from a cDNA spleen sample, and the function of IRF1 in innate immunity in geese was explored. We investigated the function of GoIRF1 in RNA virus infection and demonstrate that GoIRF1 plays a role in limiting replication and infection by NDV. Our results suggest that GoIRF1 is an important regulator of IFNs, proinflammatory cytokines, and ISGs and is involved in antiviral innate immunity in geese. These findings contribute to a more systematic understanding of the bird’s IFN-regulated signaling pathway and innate immune system and provide reference data about the general and individual characteristics of the innate immune system in birds and mammals.

## Materials and methods

### Cells and viruses

DF-1 is a chicken embryonic fibroblast cell line from East Lansing strain eggs. Goose embryonic fibroblasts (GEFs) were prepared from 15-day-old embryonated goose eggs. The DF-1 cells and GEFs were maintained in high-glucose Dulbecco's Modified Eagle's Medium (DMEM) (Corning, USA) containing 10% fetal bovine serum (FBS) (Gibco, USA) and 1% penicillin–streptomycin (Gibco). All cells were incubated at 37 °C in a 5% carbon dioxide incubator. The NDV strain NSD14 was isolated from chickens at a farm in Shandong Province, China. GFP-tagged NDV low virulent strain LaSota, named NDV-GFP and VSV-GFP, are stored in our laboratory. These three viruses were purified, propagated, and stored as described in our previous study [37].

### Cloning and bioinformatics analysis of GoIRF1

Based on the predicted GoIRF1 sequence (XM_013175976.1) obtained from the National Center for Biotechnology Information (NCBI), the primers GoIRF1-F and GoIRF1-R (Table 1), which are located outside the GoIRF1 open reading frame, were designed. The above primers and ApexHF HS DNA Polymerase FS Master Mix AG12202 (Accurate Biotechnology Co., Ltd., Hunan, China) were used to amplify GoIRF1 cDNA from a spleen sample via transcriptase polymerase chain reaction (PCR). The PCR product was ligated into a pTOPO-Blunt vector (Aidlab Biotech, Beijing, China) for sequencing, and positive colonies were sent to the Beijing Genomics Institute (Beijing, China) for sequencing. The amino acid sequence of GoIRF1 was aligned with other animal IRF1 proteins from chickens, humans, pigs, and mice using Clustal W and edited with ESPript 3.0 [38]. Sequence homology and a phylogenetic analysis of the IRF1 amino acid sequences were conducted using DNASTAR. A phylogenetic tree was constructed based on the IRF1 from 13 different species, including mammals, birds, and fish. Conserved domains in the GoIRF1 amino acid sequences were predicted using the simple modular architecture research tool (SMART) program [39]. Homology modeling for GoIRF1 was conducted using the online protein-modeling server SWISS-MODEL [40].

**Table 1:** **PCR primers used in the study**

Target gene	Purpose	Name	Sequence of oligonucleotide (5′–3′)
GoIFN-α	qRT-PCR	qGoIFN-α F	CTCCAGCACCTCTTCGACAC
		qGoIFN-α R	GTTGATGCCGAGGTGAAGGT
GoIFN-γ	qRT-PCR	qGoIFN-γ F	ACATCAAAAACCTGTCTGAGCAGC
		qGoIFN-γ R	AGGTTTGACAGGTCCACGAGG
GoIFN-κ	qRT-PCR	qGoIFN-κ F	ACAGCAAAGAAAAGTGATTG
		qGoIFN-κ R	GTTGGAAGATCTCTTCAATGG
GoIFN-λ	qRT-PCR	qGoIFN-λ F	GAGCTCTCGGTGCCCGACC
		qGoIFN-λ R	CTCAGCGGCCACGCAGCCT
GoIL-6	qRT-PCR	qGoIL-6 F	AGCAAAAAGTTGAGTCGCTGTGC
		qGoIL-6 R	TAGCGAACAGCCCTCACGGT
GoIL-8	qRT-PCR	qGoIL-8 F	GCTGTCCTGGCTCTTCTCCTGATT
		qGoIL-8 R	GGGTCCAAGCACACCTCTCTGTTG
GoPKR	qRT-PCR	qGoPKR F	GCAACAGCAAAGACTGACGA
		qGoPKR R	TGTTTGTGACCTCTGCCTTG
GoOASL	qRT-PCR	qGoOASL F	CAGCGTGTGGTGGTTCTC
		qGoOASL R	AACCAGACGATGACATACAC
GoMx-1	qRT-PCR	qGoMx-1 F	TTCACAGCAATGGAAAGGGA
		qGoMx-1 R	ATTAGTGTCGGGTCTGGGA
GoIRF1	qRT-PCR	qGoIRF1 F	TGAGAAAGACCCTGACCCCA
		qGoIRF1 R	GCTGGAGCCTTTGTTGATGC
	To obtain sequence	GoIRF1 F	CTCTGCTTCTGTCACAGCCAC
		GoIRF1 R	CTGCAGAAGCCAGGGGTTTAC
	Construction of GoIRF1	pcDNA3.1-Flag *Eco*R I	tagtccagtgtggtggaattcATGCCCGTCTCCAGAATGCG
		pcDNA3.1-Flag *Xho* I	gtcgtccttgtagtcctcgagCAAGCCACAGGAGATGGTTTG
	Construct truncated forms of GoIRF1	GoIRF1 d1-10 aa F	gtgtggtggaattcatgTGGCTGGAAATGCAG
		GoIRF1 d1-10 aa R	CATGAATTCCACCACAC
		GoIRF1 d1-50 aa F	gtgtggtggaattcatgGATGCCTGCCTTTTC
		GoIRF1 d1-50 aa R	CATGAATTCCACCACAC
		GoIRF1 d51-114 aa F	ctgggacatggagaaaTTGACAAAGGATCAG
		GoIRF1 d51-114 aa R	TTTCTCCATGTCCCAG
		GoIRF1 d120-220 aa F	GCTTCATCCTCGGAAG
		GoIRF1 d120-220 aa R	cttccgaggatgaagcCTGATCCTTTGTC
		GoIRF1 d201-317 aa F	gactggaggacgccgCTCGAGGACTACAAG
		GoIRF1 d201-317 aa R	CGGCGTCCTCCAGTC
		GoIRF1 d251-317 aa F	caggactggcacacgCTCGAGGACTACAAG
		GoIRF1 d251-317 aa R	CGTGTGCCAGTCCTG

### Plasmid construction

Flag-tagged GoIRF1 plasmids (pcDNA3.1-GoIRF1-Flag) were constructed by inserting the full-length GoIRF1 into the *Xho* I and *Eco*R I sites pcDNA3.1-Flag of the expression vector using a ClonExpress II One-Step Cloning Kit (Vazyme Biotech Co., Ltd., Nanjing, China). The primers used in the PCR are listed in Table 1. The truncated plasmids of GoIRF1, including deletion of amino acids 1 to 10 (d1-10 aa), d1-50 aa, d51-114 aa, d120-220 aa, d201-317 aa, and d251-317 aa, were constructed using a modified homologous recombination method and the primers listed in Table 1. The chicken IFN-β (chIFN-β) promoter luciferase reporter plasmids (pGL-chIFN-β-Luc), which contained − 158 to + 14 of the IFN-β promotor motif, were constructed as described in our previous study [41]. The Trelief™ 5α Chemically Competent Cell (Tsingke Biological Technology, Beijing, China) was used for plasmid transformation.

### Luciferase reporter assays

The DF-1 or GEF cells were plated in 24-well plates (NEST Biotechnology, Wuxi, China) and cultured to 95–100% confluence; they were then transiently transfected with reporter plasmid pGL-chIFN-β-Luc (0.12 µg/well) and internal control Renilla luciferase (pRL-TK, 0.06 µg/well), along with the indicated plasmids, using Nulen PlusTrans™ Transfection Reagent (Nulen, Shanghai, China). The cells were lysed 24 h after transfection, and luciferase activity was detected using the Dual-Luciferase Reporter Assay System (Promega, USA) according to the manufacturer’s instructions. Renilla luciferase activity was used for normalization. All reporter assays were repeated at least three times.

### RNA extraction and quantitative real-time PCR

After the RNA was extracted from GEFs using an HP Total RNA kit (Omega, USA), the RNA was reverse-transcribed to cDNA using a cDNA synthesis kit (Vazyme). Quantitative real-time PCR (qRT-PCR) tests were conducted using the primers listed in Table 1 and an ABI 7500 RT-PCR system. The qRT-PCR test was performed according to the manufacturer’s instructions using a ChamQTM SYBR^®^ qPCR Master Mix (Vazyme). The conditions and data processing method for the qRT-PCR test were the same as in our previous study [41].

### Western blot analysis

The DF-1 cells were plated in 12-well plates at 1 × 10^6^/1 mL and then transfected with empty plasmids or various expression plasmids. After 24 h, the transfected cells were washed twice with phosphate buffer saline (PBS) (Gibco) and then lysed with a cell lysis buffer (Beyotime, Shanghai, China) containing an InStab™ protease cocktail (Yeasen, Shanghai, China) and phenylmethylsulfonyl fluoride (PMSF) (Yeasen). The cell lysates were centrifuged at 13 000 rpm for 15 min to obtain the supernatant, and a 5 × SDS-PAGE protein loading buffer (Yeasen) was added. The lysates were then boiled for 10 min. The proteins isolated from the cell lysates were separated via SDS-PAGE and analyzed using a Western blot. Images were obtained using the Tanon 5200 imaging system (Tanon, Shanghai, China), as described in our previous study [42].

### Viral infection

The GEF cells were plated, washed twice with PBS (Gibco), and infected at 0.1 multiplicity of infection (MOI) with NSD14. The RNA from the cells, which was infected with the virus at different times, was then collected for quantitative real-time PCR (qRT-PCR). The GoIRF1-overexpressing and normal DF1 cells were infected at 0.1 MOI with NDV-GFP or VSV-GFP, and fluorescence was measured 24 h after infection using a fluorescence microscope.

### Statistical analysis

The data were expressed as means ± standard deviations. Significance was determined using a two-tailed independent Student’s *t* test (*p < 0.05, **p < 0.01, ***p < 0.001).

## Results

### Cloning and sequence analysis of GoIRF1

**Figure 1:**
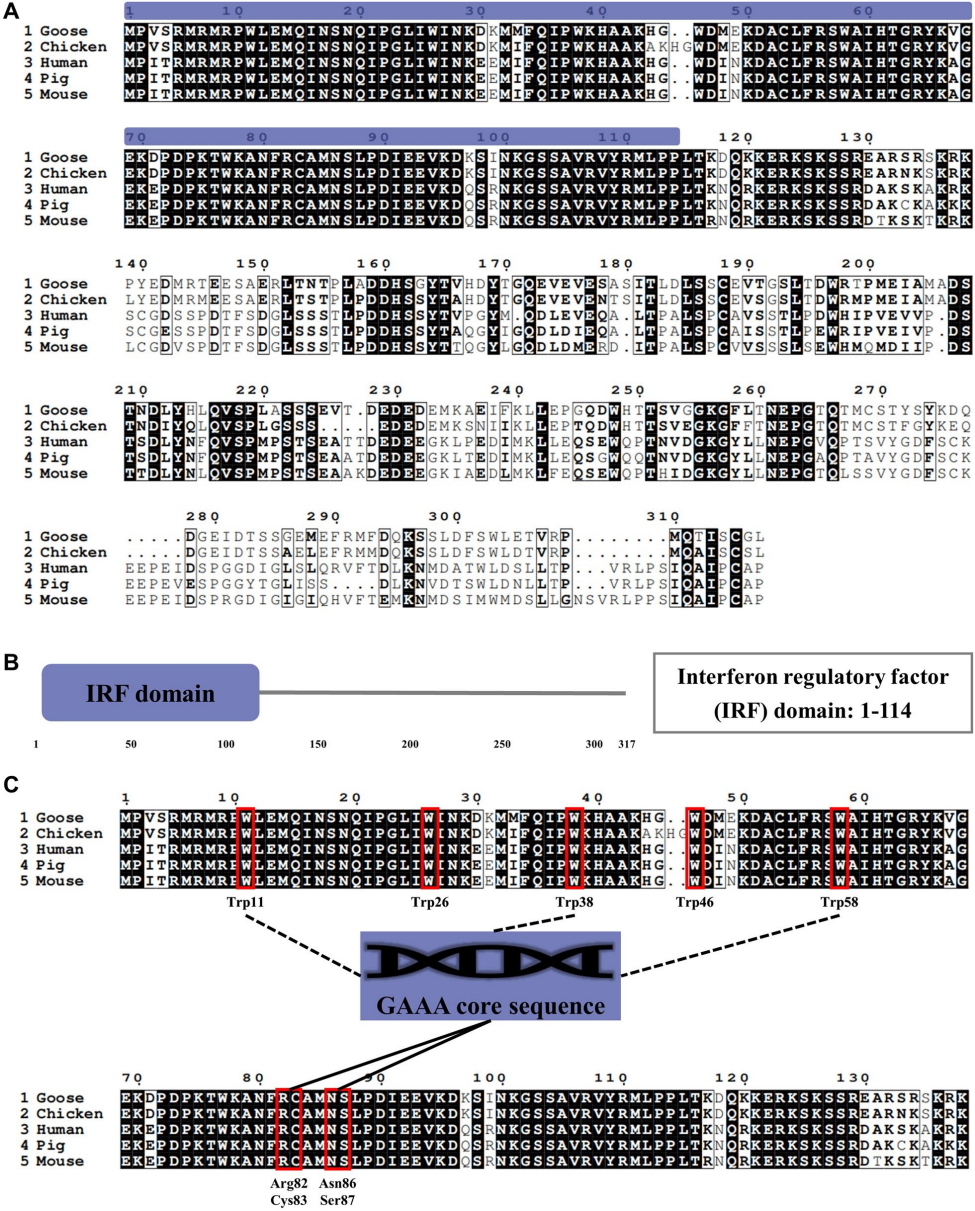
**Sequence analysis of GoIRF1.** **A** Alignment of the amino acid sequence of GoIRF1 with IRF1 proteins from humans (NP_002189.1), mice (CAJ18442.1), pigs (NP_001090882.1), and chickens (NP_990746.1). The amino acid sequences of different animals were aligned using Clustal W and edited with ESPript 3.0. The black shading indicates the identity of the amino acid, and the gray shading indicates similarity (50% threshold). **B** Protein domains of GoIRF1 predicted by SMART. **C** The elements that mediate 5′-GAAA-3′ sequence recognition in IRF1 are well preserved among different species.

To better understand the role of GoIRF1 in innate immunity in geese, we cloned GoIRF1 and conducted a bioinformatics analysis. The open reading frame of GoIRF1 contains 954 bp and encodes 317 amino acid residues. The IRF1 amino acid sequences in chickens (Gallus gallus, NP_990746.1), humans (Homo sapiens, NP_002189.1), mice (Mus musculus, CAJ18442.1) and pigs (Sus scrofa, NP_001090882.1) are 90.1%, 62.2%, 60.1.4%, and 59.6% identical to those in geese, respectively (Figure 1A). The protein domains of GoIRF1 were predicted using SMART. The results show that there is an IRF domain (DBD) at the N-terminal of GoIRF1 (aa 1-114) (Figure 1B). The DBD of IRF1 in geese and mammals is highly conserved. In particular, the elements (W11, W38, W58, R82, C83, N86, S87) that mediate 5′-GAAA-3′ sequence recognition are well preserved among different species (Figure 1C).

### Phylogenetic tree analyses and the three-dimensional structure of GoIRF1

**Figure 2:**
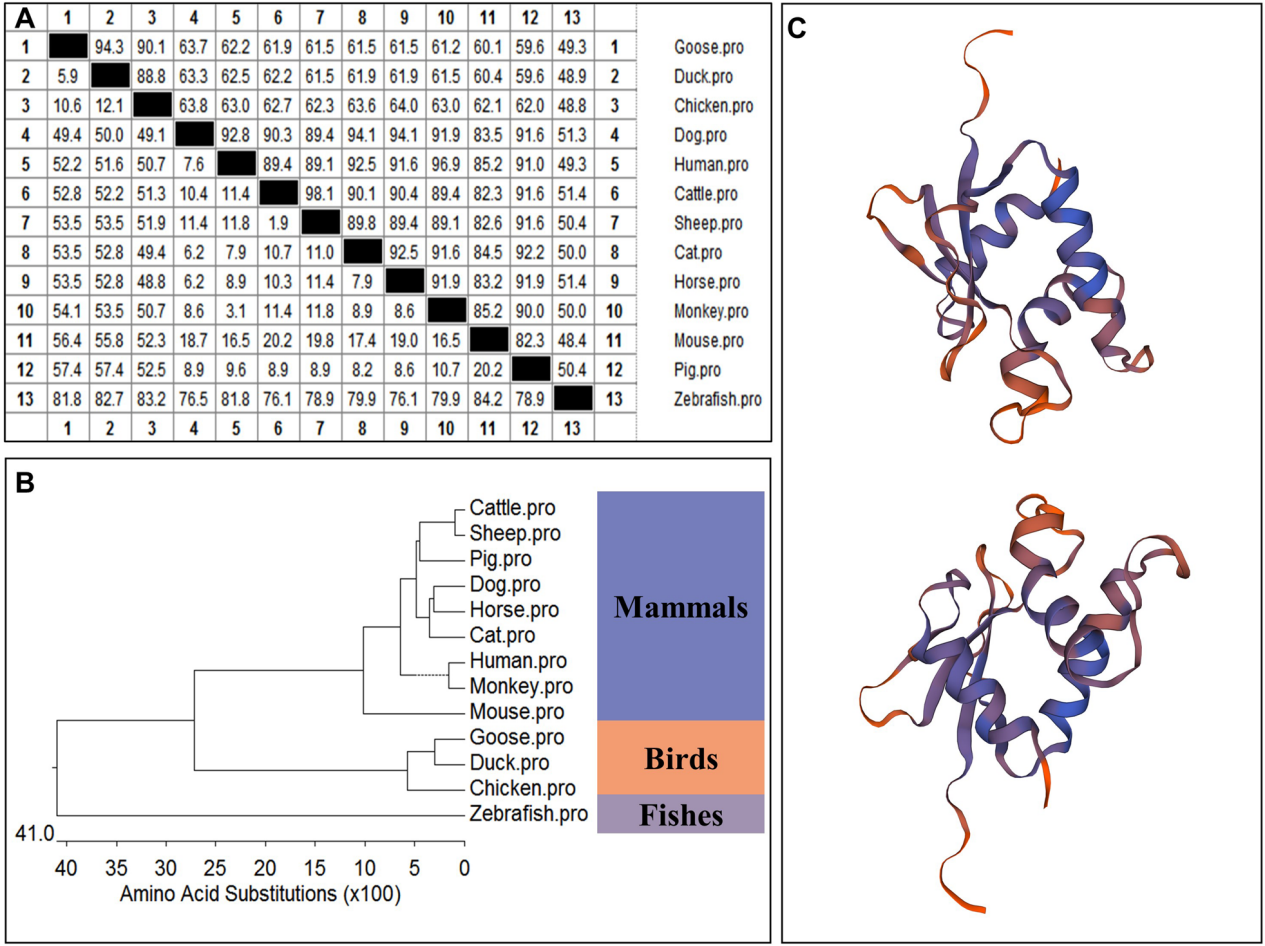
**Phylogenetic tree analyses and the three-dimensional structure of GoIRF1.** **A** The amino acid sequence homology of different animals. **B** Phylogenetic tree of vertebrate IRF1. A neighbor-joining phylogenetic tree of vertebrate IRF1 was generated with MegAlign software using IRF1 sequences from the following animals: cattle (NP_001178190.1), sheep (NP_001009751.1), pigs (NP_001090882.1), dogs (XP_003639416.3), horses (XP_005599497.1), cats (XP_003980801.1), humans (NP_002189.1), monkeys (XP_012300198.1), mice (CAJ18442.1), chickens (NP_990746.1), ducks (XP_027324327.2), zebrafish (NP_991310.1), and geese. **C** Three-dimensional structure of GoIRF1 predicted using SWISS-MODEL.

The amino acid sequence homologies of different animals were conducted using MegAlign; the results are shown in Figure 2A. A phylogenetic tree was developed based on multiple alignments of IRF1 from various species, including mammals, birds, and fish (Figure 2B). The resulting phylogenetic tree consists of three major branches. The IRF1 protein sequences of geese, chickens (NP_990746.1), and ducks (XP_027324327.2) belong to one subgroup. The IRF1 of mammals, including cattle (NP_001178190.1), sheep (NP_001009751.1), pigs (NP_001090882.1), dogs (XP_003639416.3), horses (XP_005599497.1), cats (XP_003980801.1), humans (NP_002189.1), monkeys (XP_012300198.1), and mice (CAJ18442.1), belongs to another subgroup. IRF1 sequences from zebrafish (NP_991310.1) belong to a third subgroup. These categorizations reflect the genetic relationships among these species. The predicted three-dimensional structures of GoIRF1 are shown in Figure 2C.

### Upregulation of GoIRF1 expression in response to viral infection

**Figure 3:**
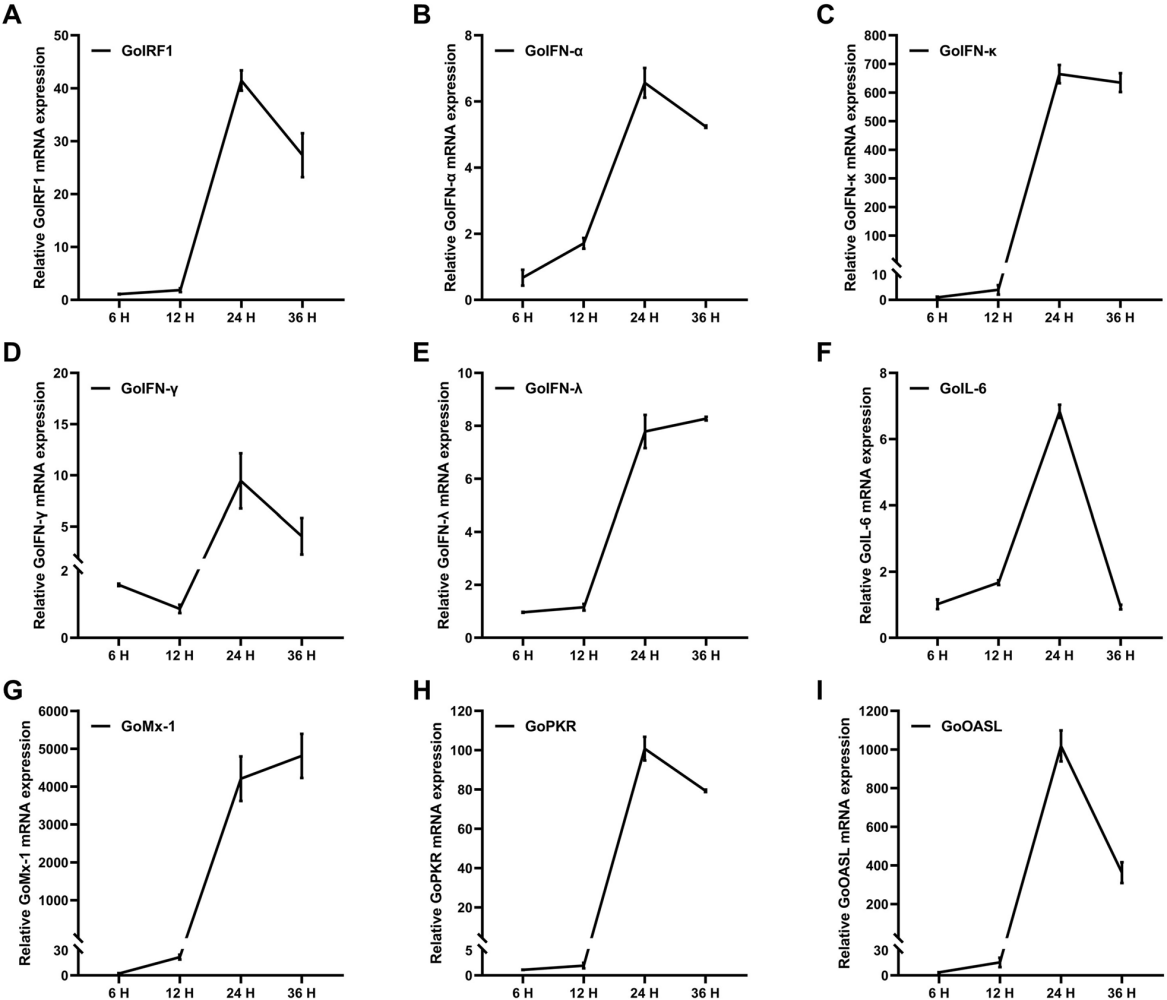
**Upregulation of GoIRF1, IFNs, IL-6, and ISGs expression in response to viral infection.** **A** Upregulation of goose IRF1 in GEFs infected with NDV at 0.1 MOI. **B**–**E** Upregulation of IFNs (IFN-α, IFN-κ, IFN-γ, and IFN-λ) in GEFs infected with NDV at 0.1 MOI. **F** Upregulation of IL-6 in GEFs infected with NDV at 0.1 MOI. **G**–**I** Upregulation of ISGs (Mx-1, PKR, and OASL) in GEFs infected with NDV at 0.1 MOI. Error bars represent standard deviations.

In chickens and some mammals, IRF1 is involved in type-I IFN-mediated antiviral innate immune response. However, the role of goose IRF1 in antiviral response remains unknown. For the host, upregulating the expression of certain immune-related genes is an important strategy in infection resistance. To determine whether GoIRF1 could induce an antiviral response to infection with NDV, we conducted a preliminary analysis of the expression of GoIRF1, of some cytokines, and of the IFN-stimulated genes in GEF cells following infection with NDV. The results show that the mRNA levels of GoIRF1 were significantly upregulated during viral infection (Figure 3A). The mRNA levels of IFNs (IFN-α, IFN-κ, IFN-γ, and IFN-λ) (Figures 3B–E), IL6 (Figure 3F), and IFN-stimulated genes (ISGs) (Mx-1, PKR, and OASL) (Figures 3G–I) were significantly upregulated as well. These upregulations play a key role in cell resistance to exogenous insults.

### GoIRF1 involvement in the regulation of IFNs, proinflammatory cytokines, and ISGs

**Figure 4:**
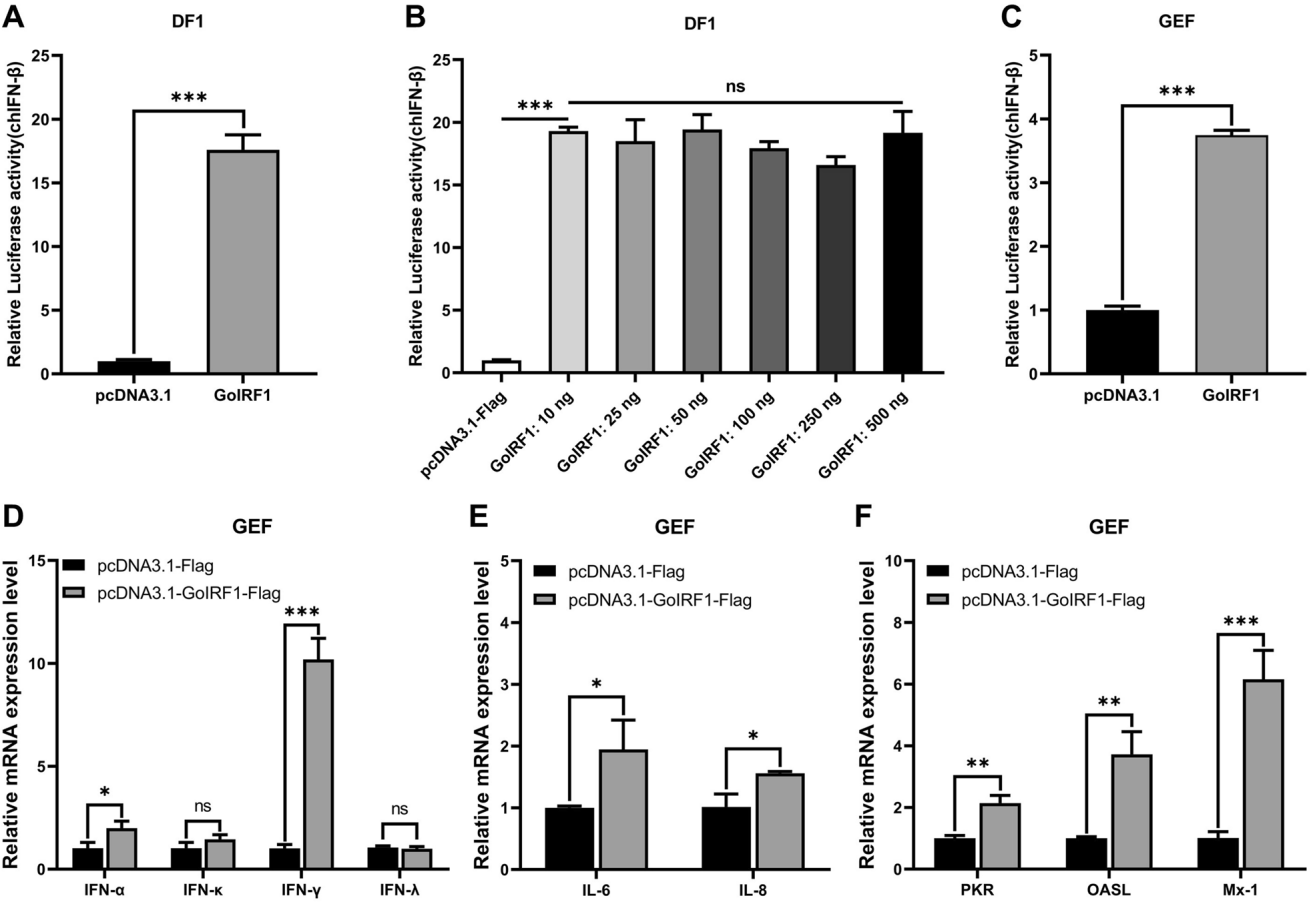
**Goose IRF1 is involved in the regulation of IFNs, proinflammatory cytokines, and ISGs.** **A** DF-1 cells were cotransfected with chIFN-β luciferase reporter plasmids (pRL-TK and pGL-chIFN-β-Luc) and with pcDNA3.1-Flag or pcDNA3.1-GoIRF1-Flag. Luciferase assays were performed 24 h after cotransfection. **B** GoIRF1 dose-independently induced IFN-β induction. **C** GEFs were cotransfected with chIFN-β luciferase reporter plasmids and with pcDNA3.1-Flag or pcDNA3.1-GoIRF1-Flag. Luciferase assays were performed 24 h after cotransfection. **D** Endogenous mRNA levels of goose IFNs (IFN-α, IFN-κ, IFN-γ, and IFN-λ) after stimulation with GoIRF1 or an empty vector. **E** Relative mRNA levels of proinflammatory cytokines (IL-6 and IL-8) after transfection with GoIRF1 or an empty vector. **F** Relative mRNA levels of ISGs (PKR, OASL, and Mx-1) after transfection with GoIRF1 or an empty vector. Error bars represent standard deviations. The difference between the experimental and control groups was *p < 0.05, **p < 0.01 or ***p < 0.001.

IRF1 plays an important role in mammalian antiviral innate immunity, but its function in geese is unknown. To investigate whether GoIRF1 is also involved in the regulation of IFN production, DF-1 cells were cotransfected with GoIRF1 expression plasmids and with chIFN-β luciferase reporter plasmids. We found that the overexpression of GoIRF1 in DF-1 cells significantly activated the chIFN-β promoter (Figure 4A), and this activation is independent of the dosage of the transfected GoIRF1 plasmids (Figure 4B). To further confirm the role of GoIRF1 in IFN activation, we conducted luciferase assays with primary goose embryonic fibroblasts (GEFs). This test also shows that the overexpression of GoIRF1 in GEFs significantly activated the chIFN-β promoter (Figure 4C). To further explore the effect of GoIRF1 on the expression of innate immune genes, we conducted qRT-PCR tests after we transfected GoIRF1 or an empty vector into GEFs. The results show that the mRNA levels of IFNs (IFN-α and IFN-γ), proinflammatory cytokines (IL-6 and IL-8), and IFN-stimulated genes (PKR, OASL, and Mx-1) increased significantly when GoIRF1 was overexpressed (Figures 4D–F). In the presence of overexpressed GoIRF1, the expression levels of IFN-α, IFN-γ, IL-6, IL-8, PKR, OASL, and Mx-1 were 2.0, 10.2, 1.9, 1.6, 2.1, 3.7, and 6.2 times higher than in the controls (which were transfected with an empty vector), respectively. In addition, the overexpression of GoIRF1 in GEFs did not induce the upregulation of IFN-κ and IFN-λ.

### Essential domains of GoIRF1 in IFN activation

**Figure 5:**
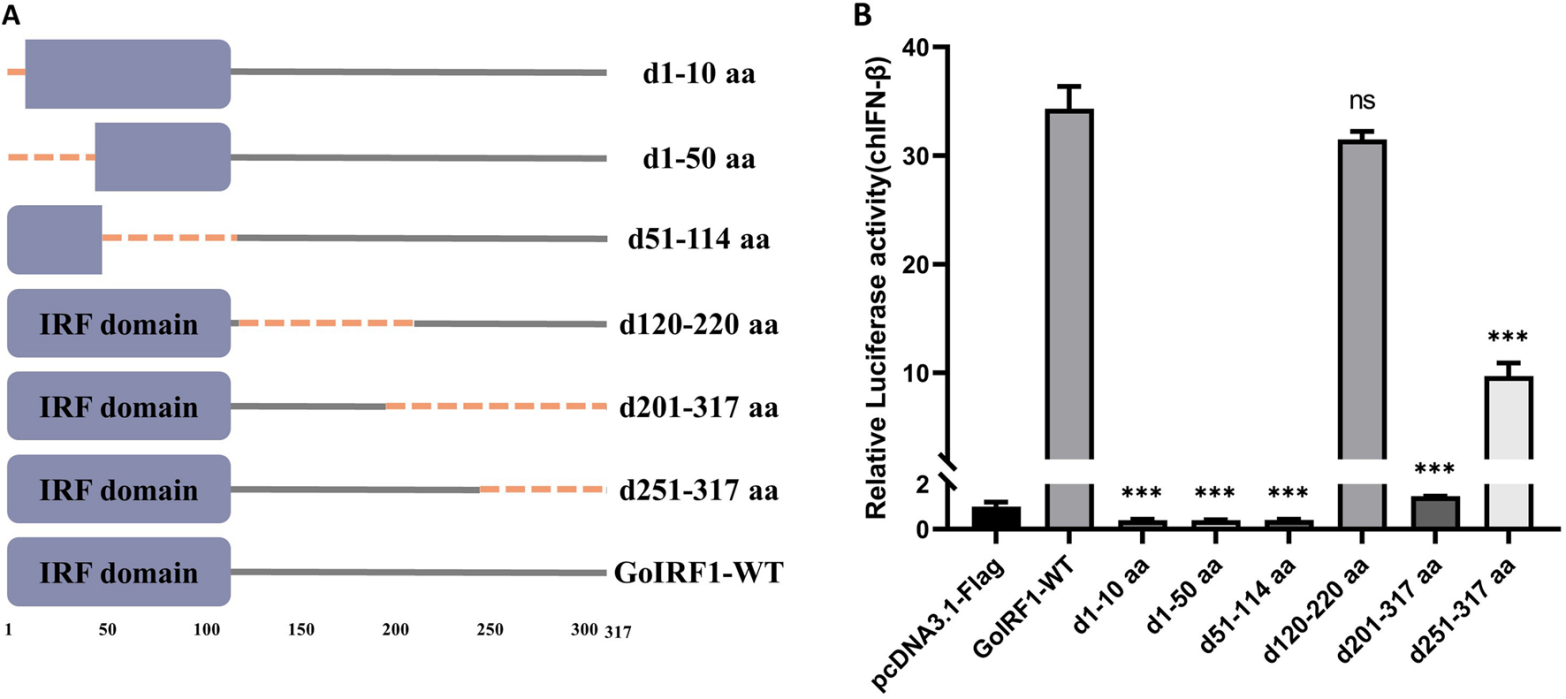
**GoIRF1 domains that are essential to IFN-β promoter activity.** **A** Schematic structure of GoIRF1 mutants. **B** The effects of GoIRF1 truncated mutants on IFN-β promoter activity. Error bars represent standard deviations. The difference between the experimental and control groups was ***p < 0.001.

The secondary structure predicted by the SMART program shows that there is an IRF domain (DBD) at the N-terminal of GoIRF1 (aa 1-114). A series of truncated forms of GoIRF1 were generated based on the secondary structure of GoIRF1. The ability of these forms to activate the IFN-β promoter was measured and compared to that of wild-type GoIRF1 using the dual luciferase reporter gene detection method (Figures 5A and B). As shown in Figure 5B, the deletion of 10 (d1-10 aa) or 50 (d1-50 aa) amino acids at the GoIRF1 N-terminal clearly decreased IFN-β promoter activity. Compared with the wild type, GoIRF1 has a significantly reduced ability to activate IFN-β when amino acids 51–114 are deleted. The deletion of amino acids 120–220 had little effect on IFN-β activation by GoIRF1. The C-terminal deletion mutants of GoIRF1 (d201-317 aa and d251-317 aa) also led to a significant degradation in IFN-β promoter activation. These results indicate that the first 114 amino acid sequences of GoIRF1 contain domains that are pivotal for inducing IFN-β promoter activity. In addition, the deletion of the C-terminal amino acid of GoIRF1 also led to a significant degradation in IFN-β promoter activation.

### The in vitro antiviral role of GoIRF1

**Figure 6:**
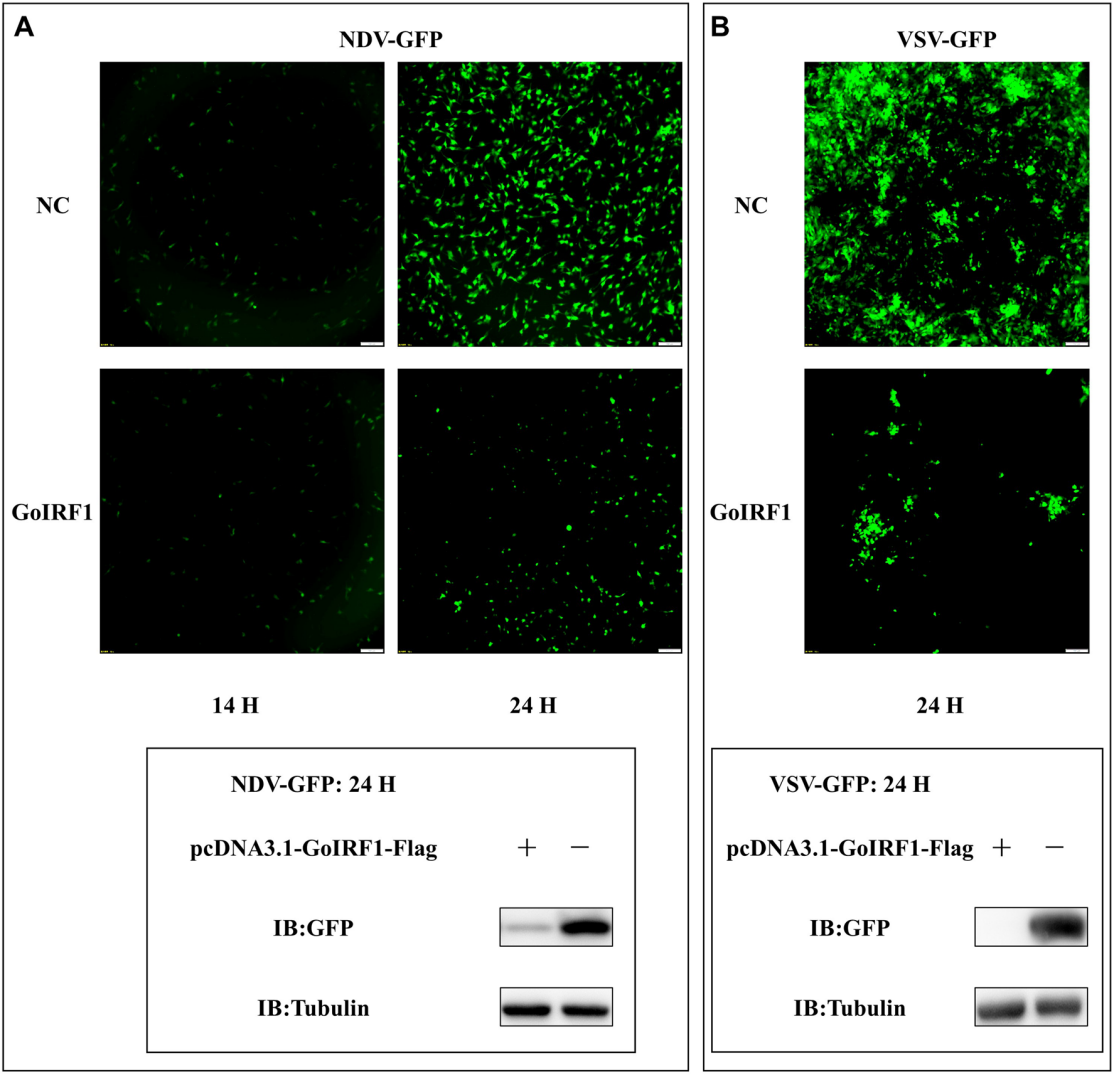
**GoIRF1 inhibits viral yield.** **A** Viral fluorescence in cells transfected with pcDNA3.1-Flag or pcDNA3.1-GoIRF1-Flag and infected with NDV-GFP at 0.1 MOI. GFP-tagged NDV-GFP in cells 24 h after infection was quantified using Western blotting, and immunodetection was measured using anti-GFP Ab. **B** Viral fluorescence in cells transfected with pcDNA3.1-Flag or pcDNA3.1-GoIRF1-Flag and infected with VSV-GFP at 0.1 MOI. GFP-tagged VSV-GFP in cells 24 h after infection was quantified using Western blotting, and immunodetection was measured using anti-GFP Ab.

To test the antiviral effects of GoIRF1, the GoIRF1-overexpressing and normal DF-1 cells were infected with VSV-GFP or NDV-GFP, and fluorescence was measured with a fluorescence microscope. The fluorescence intensities of both VSV-GFP and NDV-GFP in the cells that overexpressed GoIRF1 were significantly lower than those in the control cells 14 or 24 h after viral infection (Figures 6A and B). This result suggests that GoIRF1 overexpression in DF-1 cells suppresses VSV-GFP and NDV-GFP viral replication. The virus-infected cells were then lysed and collected for Western blot analysis. The GFP protein expression levels of NDV-GFP and VSV-GFP in the cells overexpressing GoIRF1 were significantly lower than those of the controls (which were transfected with an empty vector).

## Discussion

Mammalian IRFs play a key role in many aspects of the immune response, and IRF1 is a positive mediator of IFN induction after PRRs recognize PAMPs [43]. Compared with mammals, birds have fewer immune genes, and IRF3, one of the most important IFN regulatory factors in mammals, is naturally absent in birds [15]. It is widely accepted that a closely related IRF7, considered to be the major IRF in birds, complements the functions of IRF3, and that other IRFs may compensate the function [18, 20]. It remains to be studied whether IRF3 deletion leads to deficiencies in the innate immune system of birds and whether IRF1 plays an immune regulatory function in IRF3-absent birds. Although the IRF1 of chickens and ducks has been shown to be an important regulatory factor involved in IFN regulation [35, 36], geese have shown more significant advantages in resisting AIV and NDV infections [44, 45], and IRF1 as a key IFN regulatory factor may be the cause of the difference in disease resistance. A better understanding of the function of GoIRF1 may help explain these differences.

First, we cloned GoIRF1 and analyzed the amino acid sequences of IRF1 from different animals. The N-terminus of IRF1 in different animals contains a DBD, which is highly conserved among different species, while the C-terminus has poor homology, which determines the uniqueness of its function (Figure 1A). The conserved domains predicted by SMART show that there is an IRF domain (DBD) at the N-terminal of GoIRF1 (aa 1-114) (Figure 1B). Not surprisingly, the DBD of IRF1 in geese and mammals is highly conserved. In particular, the elements (W11, W38, W58, R82, C83, N86, and S87) that mediate 5′-GAAA-3′ sequence recognition are well preserved among geese and mammals (Figure 1C). These conserved elements allow GoIRF1 to bind to the core sequence on the PRD1 of the IFN promoter to regulate IFN production [27]. The resulting phylogenetic tree consists of three major branches (Figures 2A and B). The IRF1 protein sequences of geese, ducks, and chickens belong to one subgroup. The IRF1 of mammals, including humans, monkeys, mice, cats, horses, dogs, cattle, sheep, and pigs, belong to another subgroup. IRF1 sequences from zebrafish belong to a third subgroup. These results reflect the genetic relationships among these species. The predicted three-dimensional structures of GoIRF1 are shown in Figure 2C.

IRF1 was initially identified as a positive regulator that directly binds to the promoter of type I IFN genes, but subsequent studies have found that the absence of IRF1 in mice does not affect the induction of IFN-β [46]. Whether IRF1 is a necessary IFN regulatory factor was once in controversy. With the deepening of innate immunity research, IRF1 was found to interact with MyD88 to control the production of TLR9-dependent IFN-β in mouse myeloid dendritic cells [47]. IRF1 also plays a positive regulatory role in the innate immune response to viral infection by enhancing the phosphorylation of IRF3 and the production of types I and III IFN triggered by viral infection [48]. For the host, upregulating the expression of certain immune-related genes is a required strategy in infection resistance. Mammalian IRF1 is upregulated by viral stimulation and resists viral infection by regulating IFNs and ISGs [48]. A qRT-PCR test shows that GoIRF1 mRNA was significantly upregulated in GEFs infected with NDV. Some key cytokines and ISGs that resist viral infection are also significantly upregulated, showing the same expression trend as IRF1. These results indicate that GoIRF1 may be involved in the regulation of antiviral innate immunity in geese. In cell experiments, we found that the overexpression of GoIRF1 in GEFs or DF-1 cells strongly induced the expression of chIFN-β, and this induction is independent of the amount of GoIRF1 plasmid transfected (Figures 4A–C). Other types of IFNs, including IFN-α and IFN-γ, were also activated by GoIRF1 overexpression, while IFN-κ and IFN-λ were not upregulated (Figure 4D). In addition, the overexpression of GoIRF1 in GEF cells significantly upregulated the expression of several proinflammatory cytokines (IL-6 and IL-8) and IFN-stimulated genes (PKR, OASL, and Mx-1) (Figures 4E and F). Our results indicate that GoIRF1 positively regulates the expression of IFNs, proinflammatory cytokines, and ISGs.

To identify the domain of GoIRF1 that is indispensable to IFN induction, a series of truncated forms of GoIRF1 were generated, and their abilities to induce IFN-β promoter activity were compared (Figure 5). The deletion of different fragments of the IRF domain (d1-10 aa, d1-50 aa, and d51-114 aa) leads to the loss of its function of activating IFN-β. These mutated IRF1 may be due to its inability to recognize the 5′-GAAA-3′ core sequence on the IFN-β promoter, leading to the loss of its function, which indicates that the complete DBD is necessary for GoIRF1 to activate IFN-β. The deletion of amino acid fragments of different sizes at the C-terminus of GoIRF1 also results in a significant decrease in its ability to activate the IFN-β promoter. In contrast, the deletion of amino acids 120–220 has little effect on the activation of the IFN-β promoter by GoIRF1. Our previous studies on the indispensable domains of chicken IRF1 to activate IFN also showed the same characteristics [35].

IRF1 plays an important role in the antiviral innate immunity of mammals, but the research on its antiviral function in birds is incomplete. In particular, the antiviral function of goose IRF1 is unknown. To further verify the immunomodulatory and antiviral activities of GoIRF1, we conducted a series of experiments to explore the effect of GoIRF1 on virus replication. The results suggest that GoIRF1 overexpression in DF-1 cells significantly suppresses the viral replication of VSV-GFP and NDV-GFP (Figure 6).

In brief, our results show that GoIRF1 is an important regulator of IFNs, proinflammatory cytokines, and ISGs and is involved in goose antiviral innate immunity. Our findings contribute to a more systematic understanding of the innate immune system of geese and could help explain the differences in the resistance of different birds to viral infection. Our results also provide reference data about the general and individual characteristics of innate immunity in birds and mammals.

## Data Availability

The datasets used and analysed during the current study are available from the corresponding author on reasonable request.

## References

[1] Jensen S, Thomsen AR (2012). Sensing of RNA viruses: a review of innate immune receptors involved in recognizing RNA virus invasion. J Virol.

[2] Akira S, Uematsu S, Takeuchi O (2006). Pathogen recognition and innate immunity. Cell.

[3] Wen H, Miao EA, Ting JP (2013). Mechanisms of NOD-like receptor-associated inflammasome activation. Immunity.

[4] O’Neill LA, Golenbock D, Bowie AG (2013). The history of Toll-like receptors - redefining innate immunity. Nat Rev Immunol.

[5] Yoneyama M, Kikuchi M, Natsukawa T, Shinobu N, Imaizumi T, Miyagishi M, Taira K, Akira S, Fujita T (2004). The RNA helicase RIG-I has an essential function in double-stranded RNA-induced innate antiviral responses. Nat Immunol.

[6] Ma Z, Ni G, Damania B (2018). Innate sensing of DNA virus genomes. Annu Rev Virol.

[7] Takeuchi O, Akira S (2009). Innate immunity to virus infection. Immunol Rev.

[8] Liu G, Gack MU (2020). Distinct and orchestrated functions of RNA sensors in innate immunity. Immunity.

[9] Yarovinsky F (2014). Innate immunity to *Toxoplasma gondii* infection. Nat Rev Immunol.

[10] Garcia-Sastre A, Biron CA (2006). Type 1 interferons and the virus-host relationship: a lesson in détente. Science.

[11] Pestka S, Krause CD, Walter MR (2004). Interferons, interferon-like cytokines, and their receptors. Immunol Rev.

[12] Honda K, Takaoka A, Taniguchi T (2006). Type I interferon [corrected] gene induction by the interferon regulatory factor family of transcription factors. Immunity.

[13] Savitsky D, Tamura T, Yanai H, Taniguchi T (2010). Regulation of immunity and oncogenesis by the IRF transcription factor family. Cancer Immunol Immunother.

[14] Huang B, Qi ZT, Xu Z, Nie P (2010). Global characterization of interferon regulatory factor (IRF) genes in vertebrates: glimpse of the diversification in evolution. BMC Immunol.

[15] Santhakumar D, Rubbenstroth D, Martinez-Sobrido L, Munir M (2017). Avian interferons and their antiviral effectors. Front Immunol.

[16] Nehyba J, Hrdlickova R, Burnside J, Bose HR (2002). A novel interferon regulatory factor (IRF), IRF-10, has a unique role in immune defense and is induced by the v-Rel oncoprotein. Mol Cell Biol.

[17] Honda K, Taniguchi T (2006). IRFs: master regulators of signalling by Toll-like receptors and cytosolic pattern-recognition receptors. Nat Rev Immunol.

[18] Kim TH, Zhou H (2015). Functional analysis of chicken IRF7 in response to dsRNA analog poly(I:C) by integrating overexpression and knockdown. PLoS ONE.

[19] Kim TH, Zhou H (2015). Correction: functional analysis of chicken IRF7 in response to dsRNA analog poly(I:C) by integrating overexpression and knockdown. PLoS ONE.

[20] Neerukonda SN, Katneni U (2020). Avian pattern recognition receptor sensing and signaling. Vet Sci.

[21] Li L, Chen SN, Nie P (2021). IRF11 regulates positively type I IFN transcription and antiviral response in mandarin fish, *Siniperca**chuatsi*. Dev Comp Immunol.

[22] Jefferies CA (2019). Regulating IRFs in IFN driven disease. Front Immunol.

[23] Yanai H, Negishi H, Taniguchi T (2012). The IRF family of transcription factors: inception, impact and implications in oncogenesis. Oncoimmunology.

[24] Miyamoto M, Fujita T, Kimura Y, Maruyama M, Harada H, Sudo Y, Miyata T, Taniguchi T (1988). Regulated expression of a gene encoding a nuclear factor, IRF-1, that specifically binds to IFN-beta gene regulatory elements. Cell.

[25] Fujita T, Sakakibara J, Sudo Y, Miyamoto M, Kimura Y, Taniguchi T (1988). Evidence for a nuclear factor(s), IRF-1, mediating induction and silencing properties to human IFN-beta gene regulatory elements. EMBO J.

[26] Fujita T, Kimura Y, Miyamoto M, Barsoumian EL, Taniguchi T (1989). Induction of endogenous IFN-alpha and IFN-beta genes by a regulatory transcription factor, IRF-1. Nature.

[27] Escalante CR, Yie J, Thanos D, Aggarwal AK (1998). Structure of IRF-1 with bound DNA reveals determinants of interferon regulation. Nature.

[28] Carlin AF, Plummer EM, Vizcarra EA, Sheets N, Joo Y, Tang W, Day J, Greenbaum J, Glass CK, Diamond MS, Shresta S (2017). An IRF-3-, IRF-5-, and IRF-7-independent pathway of dengue viral resistance utilizes IRF-1 to stimulate type I and II interferon responses. Cell Rep.

[29] Motz C, Schuhmann KM, Kirchhofer A, Moldt M, Witte G, Conzelmann KK, Hopfner KP (2013). Paramyxovirus V proteins disrupt the fold of the RNA sensor MDA5 to inhibit antiviral signaling. Science.

[30] Feng H, Zhang YB, Gui JF, Lemon SM, Yamane D (2021). Interferon regulatory factor 1 (IRF1) and anti-pathogen innate immune responses. PLoS Pathog.

[31] Dou L, Liang HF, Geller DA, Chen YF, Chen XP (2014). The regulation role of interferon regulatory factor-1 gene and clinical relevance. Hum Immunol.

[32] Magor KE, Miranzo Navarro D, Barber MR, Petkau K, Fleming-Canepa X, Blyth GA, Blaine AH (2013). Defense genes missing from the flight division. Dev Comp Immunol.

[33] Cormican P, Lloyd AT, Downing T, Connell SJ, Bradley D, O’Farrelly C (2009). The avian Toll-Like receptor pathway–subtle differences amidst general conformity. Dev Comp Immunol.

[34] Cheng Y, Zhu W, Ding C, Niu Q, Wang H, Yan Y, Sun J (2019). IRF7 is involved in both STING and MAVS mediating IFN-beta signaling in IRF3-lacking chickens. J Immunol.

[35] Liu Y, Cheng Y, Shan W, Ma J, Wang H, Sun J, Yan Y (2018). Chicken interferon regulatory factor 1 (IRF1) involved in antiviral innate immunity via regulating IFN-beta production. Dev Comp Immunol.

[36] Qian W, Wei X, Li Y, Guo K, Zou Z, Zhou H, Jin M (2018). Duck interferon regulatory factor 1 acts as a positive regulator in duck innate antiviral response. Dev Comp Immunol.

[37] Lin Z, Wang J, Zhu W, Yu X, Wang Z, Ma J, Wang H, Yan Y, Sun J, Cheng Y (2021). Chicken DDX1 acts as an RNA sensor to mediate IFN-beta signaling pathway activation in antiviral innate immunity. Front Immunol.

[38] Robert X, Gouet P (2014). Deciphering key features in protein structures with the new ENDscript server. Nucleic Acids Res.

[39] Letunic I, Khedkar S, Bork P (2021). SMART: recent updates, new developments and status in 2020. Nucleic Acids Res.

[40] Waterhouse A, Bertoni M, Bienert S, Studer G, Tauriello G, Gumienny R, Heer FT, de Beer TAP, Rempfer C, Bordoli L, Lepore R, Schwede T (2018). SWISS-MODEL: homology modelling of protein structures and complexes. Nucleic Acids Res.

[41] Cheng Y, Sun Y, Wang H, Yan Y, Ding C, Sun J (2015). Chicken STING mediates activation of the IFN gene independently of the RIG-I gene. J Immunol.

[42] Niu Q, Cheng Y, Wang H, Yan Y, Sun J (2019). Chicken DDX3X activates IFN-beta via the chSTING-chIRF7-IFN-beta signaling axis. Front Immunol.

[43] Colonna M (2007). TLR pathways and IFN-regulatory factors: to each its own. Eur J Immunol.

[44] Yuan R, Cui J, Zhang S, Cao L, Liu X, Kang Y, Song Y, Gong L, Jiao P, Liao M (2014). Pathogenicity and transmission of H5N1 avian influenza viruses in different birds. Vet Microbiol.

[45] Li ZJ, Li Y, Chang S, Ding Z, Mu LZ, Cong YL (2010). Antigenic variation between Newcastle disease viruses of goose and chicken origin. Arch Virol.

[46] Reis LF, Ruffner H, Stark G, Aguet M, Weissmann C (1994). Mice devoid of interferon regulatory factor 1 (IRF-1) show normal expression of type I interferon genes. EMBO J.

[47] Schmitz F, Heit A, Guggemoos S, Krug A, Mages J, Schiemann M, Adler H, Drexler I, Haas T, Lang R, Wagner H (2007). Interferon-regulatory-factor 1 controls Toll-like receptor 9-mediated IFN-beta production in myeloid dendritic cells. Eur J Immunol.

[48] Wang J, Li H, Xue B, Deng R, Huang X, Xu Y, Chen S, Tian R, Wang X, Xun Z, Sang M, Zhu H (2020). IRF1 promotes the innate immune response to viral infection by enhancing the activation of IRF3. J Virol.

